# A clinical study examining the effects of dietary nitrate on urinary *N*-nitrosamines

**DOI:** 10.1016/j.ajcnut.2026.101239

**Published:** 2026-02-20

**Authors:** Catherine P Bondonno, Michaela L Sundqvist, Sujata Shinde, Kevin Croft, Jonathan M Hodgson, Mattias Carlström, Eddie Weitzberg, Jon O Lundberg

**Affiliations:** 1 Edith Cowan University, Joondalup, Australia; 2 University of Western Australia, Perth, Australia; 3 Department of Physiology, Nutrition and Biomechanics, The Swedish School of Sport and Health Sciences, Stockholm, Sweden; 4 Department of Physiology and Pharmacology, Karolinska Institutet, Stockholm, Sweden

**Keywords:** cancer, diet, nitrate, nitric oxide, nitrite, N-nitrosamines, vitamin C

## Abstract

**Background:**

Inorganic nitrate from dietary sources has raised health concerns due to its possible conversion into carcinogenic *N*-nitrosamines, leading to strict regulations on nitrate concentrations in food and drinking water.

**Objectives:**

In this study, which was a part of a larger randomized controlled trial, we evaluated urinary excretion of *N*-nitrosamines in response to daily dietary nitrate intake over a 5-wk period using 2 different forms of nitrate administration.

**Methods:**

A total of 231 participants with mild hypertension were randomly assigned into 3 groups. Group 1 (*n* = 78) consumed vegetables low in nitrate along with a placebo capsule (300 mg potassium chloride). Group 2 (*n* = 77) consumed the same low-nitrate vegetables plus a potassium nitrate supplement (300 mg). Group 3 (*n* = 77) consumed nitrate-rich leafy green vegetables providing 300 mg nitrate daily plus the placebo capsule. Twenty-four-hour urine samples were collected before and after the intervention. Nitrate was measured with high-pressure liquid chromatography and *N*-nitrosamine concentrations were quantified using ultra high-pressure liquid chromatography-tandem mass spectrometry. A paired *t*-test was used for statistical analyses.

**Results:**

As expected, urinary nitrate increased ∼5- to 6-fold in participants consuming nitrate-rich vegetables or potassium nitrate compared with those consuming potassium chloride. Total urinary excretion of *N*-nitrosamines was low across all groups under basal conditions (<5 μg/24 h) and did not significantly change after the intervention. A similar lack of change was observed for each of the 7 individual *N*-nitrosamine species measured.

**Conclusions:**

These findings suggest that a 5-wk dietary intake of nitrate mostly exceeding the current consensus for upper limit of the acceptable daily intake (3.7 mg/kg/d), whether provided as a vegetable source or as a nitrate salt, does not increase urinary excretion of *N*-nitrosamines.

This study was registered at clinicaltrials.gov as NCT02916615.

## Introduction

Public concern about nitrate and nitrite as potential carcinogens has persisted for more than half a century, fueled by scientific debate and media attention [1,2]. This originates from early studies demonstrating diethylnitrosamine-induced liver cancer in rats [3], prompting a wave of investigations into *N*-nitroso compounds (NOCs) and their carcinogenic potential. Further concern arose when it was shown that ingested nitrate is converted to nitrite in human saliva, and nitrite can react with amines to form *N*-nitrosamines, thereby indirectly linking dietary nitrate with potential carcinogenic nitrosamine formation [2,4]. Animal studies have formed the foundation for current nitrate and nitrite intake guidelines. Although early experiments indicated that nitrite combined with amines could be carcinogenic, evidence for nitrate’s carcinogenicity has remained limited and inconclusive.

In its comprehensive evaluation of the evidence, the International Agency for Research on Cancer (IARC) reviewed nitrate and nitrite in 2010 [5]. IARC concluded that when ingested under conditions that enhance endogenous nitrosation—such as concurrent intake of nitrosatable amines or low antioxidant status—nitrate and nitrite are probably carcinogenic to humans (group 2A). This classification was based on sufficient evidence of carcinogenicity from endogenous nitrosation in experimental rats and mechanistic data demonstrating the formation of carcinogenic NOCs under physiologically relevant conditions.

Epidemiologic studies have long sought to clarify the relationship between nitrate exposure through diet and drinking water and cancer risk in humans; however, consistent evidence of harm remains elusive, in particular, for dietary derived nitrate [6]. A central paradox in this field is that vegetables, widely recognized for their protective effects against major diseases, including cancer, are also the primary source of dietary nitrate. One commonly proposed explanation is that vegetables are rich in antioxidants, such as polyphenols, vitamin C, and vitamin E, which may inhibit nitrosation reactions and thereby reduce the formation of potentially carcinogenic *N*-nitrosamines [2].

In recent years, scientific interest has increasingly shifted toward the potential cardiovascular benefits of dietary nitrate [[7], [8], [9], [10]]. A growing body of evidence indicates that nitrate can lower blood pressure and improve vascular function, primarily through its stepwise reduction to nitric oxide via the nitrate–nitrite–nitric oxide pathway [11,12]. In addition, nitrate has demonstrated ergogenic effects, a property now widely exploited by athletes to enhance performance [[13], [14], [15]]. These developments have prompted a critical reassessment of the long-standing concerns surrounding dietary nitrate, particularly in relation to regulatory limits and acceptable daily intake levels, which are currently set to 3.7 mg/kg/d [16].

In light of this evolving understanding, we conducted a dietary intervention study to investigate the impact of nitrate intake at the high end of acceptable daily intake on endogenous *N*-nitrosamine formation. We measured urinary *N*-nitrosamine concentrations in participants before and after a 5-wk intervention, during which nitrate was administered either through nitrate-rich vegetables or via capsules containing an equivalent dose of inorganic nitrate salt.

## Methods

The samples obtained for this study came from the Diet and Nitric Oxide study, in which the effects of dietary nitrate on blood pressure were studied in 231 adults with prehypertension or hypertension [17]. Baseline characteristics and some previously reported findings from this cohort have been published elsewhere [17,18]. These are briefly summarized here to provide context for the new analyses presented in this study.

### Study population

The study was approved by the Stockholm regional ethics committee and conducted between 2016 and 2019 at Karolinska University Hospital. All participants gave written informed consent. The original study was prospectively registered on clinicaltrials.gov (identifier NCT02916615).

A total of 231 adults with prehypertension or hypertension, defined according to the European Society of Cardiology guidelines (age 50–70 y, systolic blood pressure 130–159 mm Hg), were recruited in Stockholm County (Supplemental Table 1 and Supplemental Figure 1). Individuals using proton pump inhibitors, organic nitrates, antibiotics, or medications for erectile dysfunction were excluded. Further details regarding the study population and inclusion and exclusion criteria are provided in the main trial publication [17].

### Study design

During a 2-wk run-in phase, all participants consumed a standardized diet and provided low-nitrate vegetable regimen (carrots, bell peppers, cherry tomatoes, and sweet corn, 75 g of each; 300 g in total) with their main meals and were instructed to avoid all other vegetables for the entire study period. At the end of the run-in period, a 24-h urine collection was performed, and participants completed a food frequency questionnaire (FFQ) prior to the start of intervention.

Participants were randomly assigned to 1 of 3 interventions lasting for 5 wks: low-nitrate vegetables + placebo pills (150 mg potassium chloride, twice daily); low-nitrate vegetables + nitrate pills (150 mg nitrate in the form of potassium nitrate, twice daily); or leafy green vegetables (pot-grown lettuce providing 150 mg nitrate, twice daily) + placebo pills. The nitrate content in the leafy green vegetables was measured 4 times each year, and the vegetable portion size given to participants was precisely adjusted to deliver a total daily intake of 300 mg (2 × 150 mg) nitrate. All pills were consumed in conjunction with the vegetable intervention and always with normal meals. Participants reported weekly whether the vegetable or pill intake deviated from the protocol. During the final week of the intervention phase, 24-h urine collection was repeated. Nitrate content in municipal drinking water in the Stockholm area during the study period was <0.1 to 3 mg/l, which is well below the 50 mg/L regulatory threshold.

The study was double-blinded for the 2 groups consuming low-nitrate vegetables plus pills and single-blinded for the group consuming high-nitrate vegetables (the study leader knew that the participants consumed placebo pills but the participants did not know this).

### Characterization of baseline dietary intake

Baseline dietary intake during the 2-wk run-in period was calculated using a 54-item semiquantitative FFQ. Participants reported habitual consumption frequencies for predefined food items, which were converted to mean weekly intakes using predefined midpoints and expressed as daily consumption (Table 1). Standard portion sizes were assigned in accordance with Nordic Nutrition Recommendations.

**TABLE 1:** Estimated dietary intake during the 2-wk run-in period by randomization group

Intervention	Placebo	Potassium nitrate	Nitrate-rich vegetables	*P*
No. of participants	77	78	76	—
Vitamin C (mg/d)	188.5 ± 21.6	182.9 ± 21.7	185.7 ± 21.7	0.88
Antioxidant index (mean = 100)	115.7 ± 19.3	111.8 ± 20.1	113.8 ± 19.7	0.87
Amine index (mean = 100)	118.1 ± 36.3	113.5 ± 37.3	115.8 ± 36.8	0.92
Fruit intake (servings/d)	2.09 ± 0.67	1.90 ± 0.81	1.96 ± 0.80	0.292
Vegetable intake (servings/d)	4.00 ± 0.00	4.00 ± 0.00	4.00 ± 0.00	—
Processed meat (servings/wk)	7.17 ± 4.25	6.73 ± 4.35	6.71 ± 3.95	0.745
Nitrate (mg/d)	<60	<60	<60	—
Nitrite (mg/d)	4.9	4.5	4.6	—

Vegetable intake was fixed by protocol (300 g/d; 4 servings/d). Nitrate excretion was measured directly in 24-h urinary collections and varied between 53 and 56 mg in the 3 groups (nonsignificant). All other parameters including nitrite were calculated from the 54-item food frequency questionnaire. Values are mean ± SD. *P* values are from one-way analysis of variance.

To reflect the run-in protocol, all vegetable items included in the FFQ were set to zero, and investigator-provided vegetables were substituted at 300 g/d for all participants. The vegetables provided consisted of an equal-weight mixture of sweet corn, cherry tomatoes, bell peppers, and carrots. Nutrient values for vitamin C were derived from the Swedish Food Composition Database maintained by the Swedish Food Agency (https://www.livsmedelsverket.se/livsmedel-och-innehall/naringsamne/livsmedelsdatabasen) and supplemented, where necessary, with data from the USDA Food Data Central database (https://fdc.nal.usda.gov).

Dietary antioxidant exposure was estimated using published data on antioxidant and polyphenol content of foods, including oxygen radical absorbance capacity-based compilations described by Prior et al. [19], and summarized as a composite antioxidant index. Biogenic amine exposure was estimated using literature-informed classifications of foods known to contain higher concentrations of histamine, tyramine, and related amines. Antioxidant and amine exposures were summarized as composite indices and normalized to a population mean of 100.

All dietary intake calculations, data transformations, and table generation were performed with computational assistance, following a predefined analytic protocol specified by the investigators.

### *N*-nitrosamines in urine

Analysis of 7 *N*-nitrosamines [*N*-nitrosodimethylamine (NDMA), *N*-nitrosomethylethylamine (NMEA), *N*-nitrosodiethylamine (NDEA), *N*-nitrosopiperdine (NPIP), *N*-nitrosopyrrolidine (NPYR), *N*-nitrosodi-*n*-propylamine, and *N*-nitrosodi-*n*-butylamine)] in 24-h collected urines were measured by ultra-HPLC-tandem mass spectrometry, as described earlier [20]. The detection and measurement of *N*-nitrosamines was carried out on a Thermo Scientific TSQ Altis Triple Quadrupole mass analyzer, equipped with a heated electrospray ionization source attached to a Vanquish ultra-performance HPLC system. *N*-nitrosamines were extracted from urine using a sorbent supported liquid extraction method and measured using mixture of deuterated internal standards (NDMA-d_6_, NDEA-d_10_, NPIP-d_10_, and *N*-nitrosomorpholine-d_8_). The concentrations of *N*-nitrosamines were calculated using linear regression analysis and expressed in ng/mL of urine. For quality control, a 24-h collected urine from a single person spiked with a known concentration of *N*-nitrosamine standards, EPA 521 mix, and mixture of deuterated internal standards was extracted under similar experimental conditions with every batch. The percentage coefficient of variation of *N*-nitrosamines measured in the quality control urines were in the range of 15% to 20%. Urine samples were kept at −80°C for 6–9 y and shipped on dry ice between Sweden and Australia in July 2025 for analysis.

### Nitrate in urine

Nitrate was measured using an HPLC system (ENO-20; EiCom) as previously described [18].

### Statistics

The primary response variable for this analysis was the change in total urinary *N*-nitrosamines (μg/24 h) from baseline to the end of the intervention. The sample size for this analysis was determined by the original randomized clinical trial, which was powered to detect differences in blood pressure, and was therefore fixed for the present secondary analysis. Based on the observed variability in change in urinary *N*-nitrosamines (pooled SD ≈ 2.1 μg/24 h), the available sample size (*n* = 76–78 per group) provided ∼80% power at a 2-sided significance level of 0.05 to detect a within-group change of ∼0.95 μg/24 h.

Within-group changes in total and individual urinary *N*-nitrosamines were analyzed using paired *t*-tests, comparing 24-h urinary excretion before and after intervention. Data are presented as individual participant values and group summaries. Prespecified subgroup analyses included comparisons between men and women using the Mann–Whitney *U* test and examined associations between urinary *N*-nitrosamines and BMI using Spearman rank correlation. All tests were 2-tailed, and a *P* value < 0.05 was considered statistically significant. Urinary nitrate excretion was summarized descriptively to assess compliance with the intervention. Distributional assumptions were assessed by visual inspection and the Shapiro–Wilk test, and because changes in urinary *N*-nitrosamines were skewed, sensitivity analyses using log-transformed values were performed and yielded comparable results. Statistical analyses were performed with GraphPad Prism (GraphPad Software).

## Results

### Urinary *N*-nitrosamines

**FIGURE 1:**
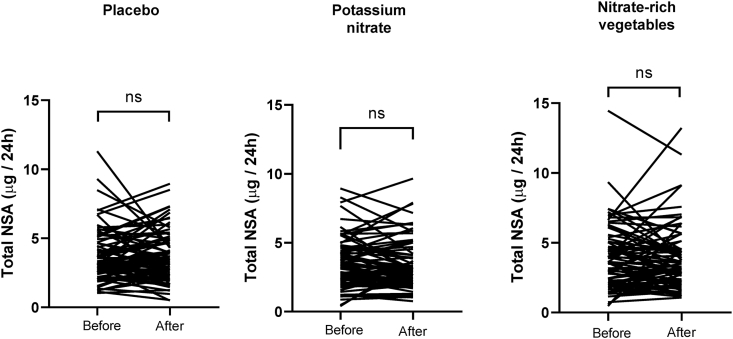
Effects of dietary nitrate on excretion of *N*-nitrosamines in urine. *N*-nitrosamines (NSA) were measured with ultra-HPLC-tandem mass spectrometry in 24-h collections of urine before and after a 5-wk dietary intervention with nitrate. Subjects were given nitrate (300 mg/d) either in the form of potassium nitrate pills (*n* = 77) or nitrate-rich vegetables (*n* = 77) and compared with a group consuming placebo pills (potassium chloride, *n* = 78). White circles represent mean; paired *t*-test was used. ns, not significant.

**FIGURE 2:**
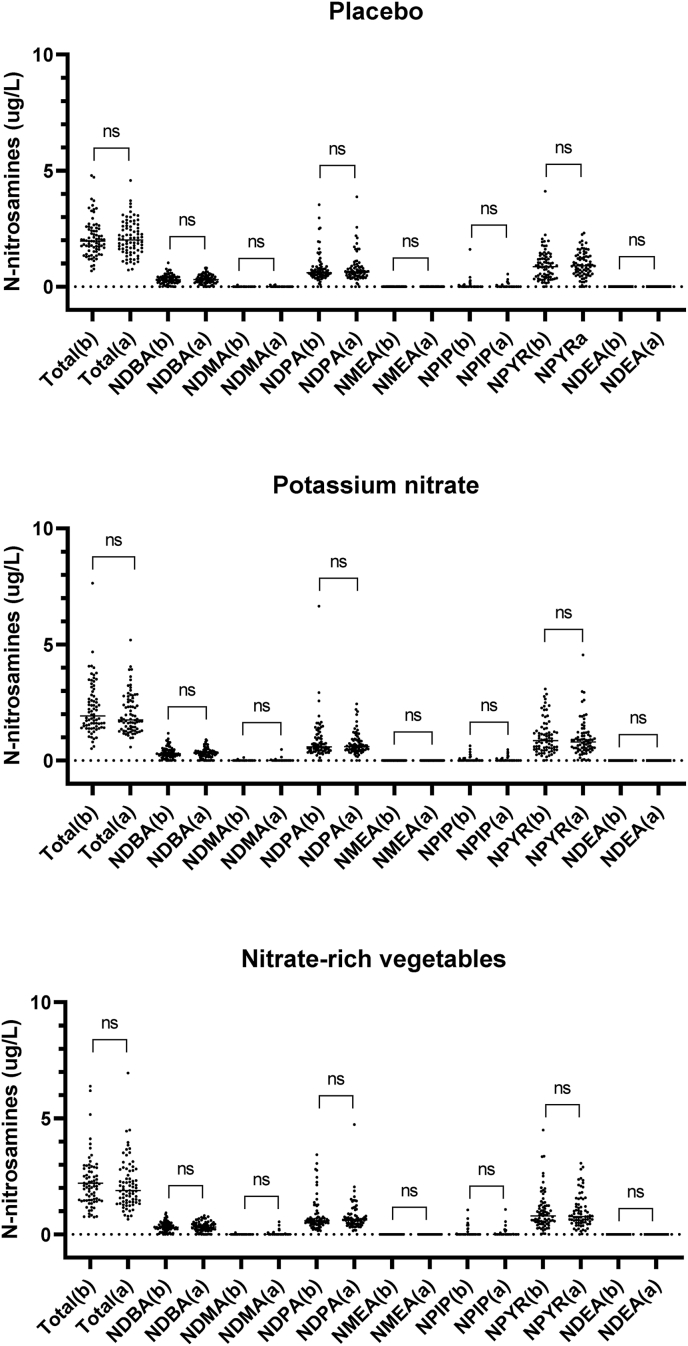
Total concentrations of *N*-nitrosamines and of 7 specific *N*-nitrosamines in urine before (b) and after (a) dietary intervention with placebo and nitrate. Paired *t*-test was used. Abbreviations: NDBA, *N*-nitrosodi-*n*-butylamine; NDEA, *N*-nitrosodiethylamine; NDMA, *N*-nitrosodimethylamine; NDPA, *N*-nitrosodi-*n*-propylamine; NMEA, *N*-nitrosomethylethylamine; NPIP, *N*-nitrosopiperdine; NPYR, *N*-nitrosopyrrolidine. ns, not significant.

**FIGURE 3:**
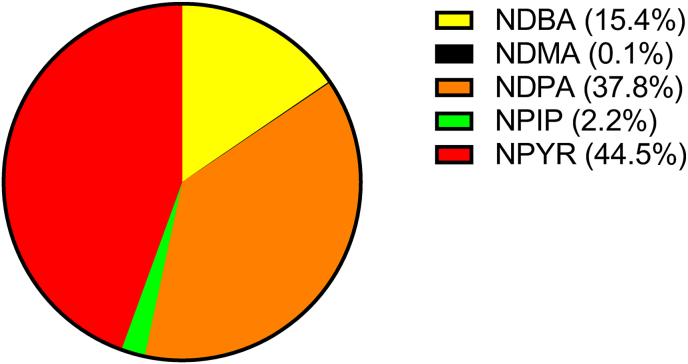
Relative proportion of 5 different *N*-nitrosamines found in urine of humans exposed to dietary nitrate for 5 wk. NMEA and NDEA were undetectable whereas NDMA is barely visible in the figure. Abbreviations: NDBA, *N*-nitrosodi-*n*-butylamine; NDMA, *N*-nitrosodimethylamine; NDPA, *N*-nitrosodi-*n*-propylamine; NPIP, *N*-nitrosopiperdine; NPYR, *N*-nitrosopyrrolidine.

Analysis of 24-h urinary excretion of total and individual *N*-nitrosamines before and after the 5-wk intervention is presented in FIGURE 1, FIGURE 2. No significant differences in total urinary *N*-nitrosamine concentrations were observed in any group (placebo, potassium nitrate, or nitrate-rich vegetables), and mean excretion was <5 μg/24 h in all groups (Figure 1). Individual participant trajectories displayed substantial overlap, and the distribution of values remained consistent pre- compared with postintervention across all groups. Similarly, no significant differences for the measured individual *N*-nitrosamines were observed between preintervention and postintervention timepoints across all intervention arms (Figure 2). Of the 7 *N*-nitrosamines measured, 2 are classed by the IARC as group 2A, probably carcinogenic to humans (NDMA and NDEA) and 5 as group 2B, possibly carcinogenic to humans (*N*-nitrosodi-*n*-propylamine, *N*-nitrosodi-*n*-butylamine, *N*-nitrosomethylethylamine, NPYR, and NPIP) [5]. NDMA and NDEA were undetectable in urine, whereas the other *N*-nitrosamines could be detected at variable concentrations (Figure 3).

Prespecified subgroup analyses showed no evidence of sex differences in urinary excretion of total *N*-nitrosamines (Mann–Whitney *U* test, *P* = 0.10) and no correlation between urinary *N*-nitrosamines and BMI (Spearman *P* = 0.016, *P* = 0.81).

### Urinary nitrate

**FIGURE 4:**
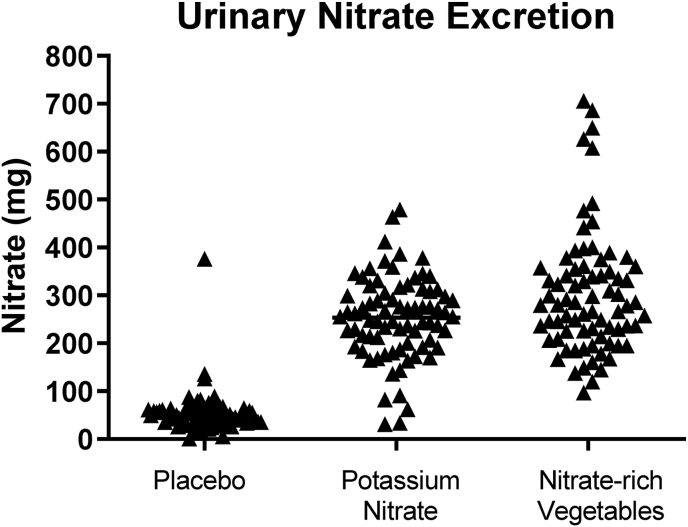
Urinary 24-h excretion of nitrate in humans exposed to nitrate. Participants were exposed to dietary nitrate (300 mg daily) for 5 wk. Nitrate was given in the form of potassium nitrate pills or nitrate-rich vegetables, and samples were compared with those from a group of participants consuming placebo (potassium chloride). Data acquired from 24-h urine collections was performed at the end of the interventions. These data have been presented previously in another context [17,18].

Urinary nitrate excretion was 5- to 6-fold higher in the groups consuming nitrate-rich vegetables (mean ± SEM, 302 ± 14.6 mg) or potassium nitrate (mean ± SEM, 254 ± 9.9 mg), whereas it remained low in the group consuming potassium chloride (mean ± SEM, 53 ± 4.9 mg, Figure 4). The close similarity in nitrate excretion between the 2 nitrate-supplemented groups demonstrates good compliance to the protocol and confirms that both groups consumed comparable amounts of nitrate regardless of source. Baseline 24-h urine collections showed that total nitrate exposure from all sources was <55 mg/24h and similar across all 3 groups [17,18]. Considering that a major portion of nitrate is also of an endogenous origin, this shows that overall intake of nitrate from food and drinking water was low in all groups at baseline.

## Discussion

Here, we demonstrate that a 5-wk dietary intervention with inorganic nitrate, administered either as a vegetable source or as a nitrate salt, markedly increases urinary nitrate excretion without elevating urinary *N*-nitrosamines in adults with prehypertension or hypertension. To our knowledge, this is the largest prospective study to date to directly examine this association, using nitrate doses at or above the current acceptable daily intake of 3.7 mg/kg [16]. These findings indicate that dietary nitrate, whether consumed as potassium nitrate or leafy green vegetables, does not promote in vivo *N*-nitrosamine formation at these intake levels. Recent clinical studies have demonstrated that inorganic nitrate can confer substantial benefits for patients with cardiovascular disease, including reductions in blood pressure and protective effects on both the heart and kidneys [11,21,22]. It is therefore plausible that doses similar to those used in this study, particularly when derived from natural sources such as leafy green vegetables, could be recommended in the near future to both healthy individuals and patients for broader cardiovascular and vascular protection. The present results indicate that such a dosing regimen does not lead to increased *N*-nitrosamine formation.

Our findings contrast with some prior reports suggesting that inorganic nitrate may increase *N*-nitrosamine formation. Berends et al. [23] recently measured apparent urinary NOCs in 29 subjects after 1 wk of daily beetroot juice consumption. They observed an acute increase in NOCs after the first dose, with a further increases after 7 d, using a slightly higher nitrate dose (400 mg/d) and a much shorter intervention period than in the present study. In addition, they quantified NOC using a nonspecific indirect assay. Interestingly, although vitamin C was able to attenuate NOC formation after the initial acute exposure, it failed to do so after 7 d of continued nitrate intake. Velmurugan et al. [24] used the same nonspecific method to quantify NOCs in 19 individuals before and after ingestion of beetroot juice. They report levels ∼2 orders of magnitude higher than those observed here, which likely reflects the limited specificity of the assay. This underscores the importance of using highly validated methods that can quantify individual *N*-nitrosamines and specifically target those of greatest relevance from a carcinogenic perspective in future work.

*N*-nitrosamines remained low and comparable between the 2 nitrate-supplemented groups after the intervention period, indicating that, at the doses used, the form of nitrate (vegetables or salt) is unlikely to influence *N*-nitrosamine formation. The notion that antioxidants in vegetables may inhibit *N*-nitrosamine formation from nitrate remains plausible because the participants consuming a nitrate salt also consumed other vegetables that are low in nitrate but still rich in antioxidants. In fact, although the specific antioxidant profiles differ between vegetable types, the total antioxidant content was likely higher in the participants consuming low-nitrate vegetables. Nevertheless, the results suggest that nitrate provided as a salt does not promote *N*-nitrosamine formation at the doses used. The timing of antioxidant intake relative to nitrate exposure may also influence *N*-nitrosamine formation and warrant investigation in future studies. Moreover, because nitrate has a relatively long half-life (∼6 h), salivary nitrite concentrations remain elevated for several hours after ingestion [6]. In the present study, nitrate supplements and vegetables were consumed in conjunction with regular meals.

This study examined nitrate intake at the upper range of typical dietary exposure but below levels used by some high-intake groups such as athletes. Future investigation should therefore also assess the safety and *N*-nitrosamine profile of substantially higher nitrate doses in these populations.

The current study has several notable strengths, including its prospective and randomized design, relatively large sample size, use of state-of-the-art technology for *N*-nitrosamine measurements, a 24-h collection of urine before and after the interventions allowing for calculation of excretion, and the exact control and matching of nitrate doses between the group consuming high-nitrate vegetable and the group consuming nitrate salt. The consistently elevated nitrate concentrations observed in urine after the intervention clearly demonstrates good compliance to the protocol and confirms that the 2 groups consumed comparable nitrate doses.

A potential limitation of this study is the prolonged storage of urine samples, albeit at −80°C, prior to analysis, which could have resulted in *N*-nitrosamine degradation. However, a comparison with earlier measurements from samples stored for a much shorter duration revealed comparable total *N*-nitrosamine concentrations [20]. Notably, both sets of measurements were conducted by the same investigators using an identical protocol and analytical method. One subject in the group consuming nitrate salt had high concentrations of NPYR in urine after the intervention. The reason for this is unknown, but because all other *N*-nitrosamines measured were in the low range in this subject, it speaks against a generally higher nitrosation level. Instead, the subject might have been exposed to preformed NPYR possibly through occasional inhalation of cigarette smoke or via food. Another limitation of the study is the sample population’s narrow age range, predominantly Caucasian ethnicity, and the presence of elevated blood pressure, which limits the generalizability of the results to the broader population. Moreover, although a 54-item FFQ completed at baseline gives some information about dietary habits, a detailed food dairy maintained during the intervention would have been preferable.

We conclude that a 5-wk intervention in adults with prehypertension or hypertension with a high dietary dose of inorganic nitrate, whether provided as nitrate-rich vegetables or as a nitrate salt, does not lead to an increase in urinary *N*-nitrosamine concentrations. Taken together with the evidence of the cardiometabolic and vascular benefits of inorganic nitrate, these findings support the safety of increasing nitrate intake in this dose range. They also provide reassurance that recommending nitrate-rich vegetables as part of a cardioprotective diet is unlikely to promote endogenous *N*-nitrosamine formation.

## Author contributions

The authors’ responsibilities were as follows – JOL, EW, MLS, CPB: designed the research; SS: analyzed the *N*-nitrosamines; JOL, EW, MLS, CPB, JMH, MC, KC: helped analyze the data, JOL, CPB: wrote the original draft; all authors: read and edited the manuscript; JOL, CPB: had primary responsibility for final content; and all authors: read and approved the final manuscript.

## Data availability

Data described in the manuscript, code book, and analytic code will be made available upon request by contacting the corresponding author JOL.

## Funding

This study was supported by grants from the Jochnick Foundation, the Swedish Research Council, Knut and Alice Wallenberg Foundation and the Swedish Heart and Lung Foundation, Axfood, funds from Karolinska Institutet, National Health and Medical Research Council Ideas Grant and Western Australian Future Health Research and Innovation Fund. Karolinska Institutet partly supported MLS’s PhD studies through the KI PhD funding system (KID).

## Conflict of interest

JOL and EW are codirectors of Heartbeet Ltd, a company holding patents for the medical uses of inorganic nitrate and nitrite. All other authors report no conflicts of interest.

## Figures and Tables

**Figure:**
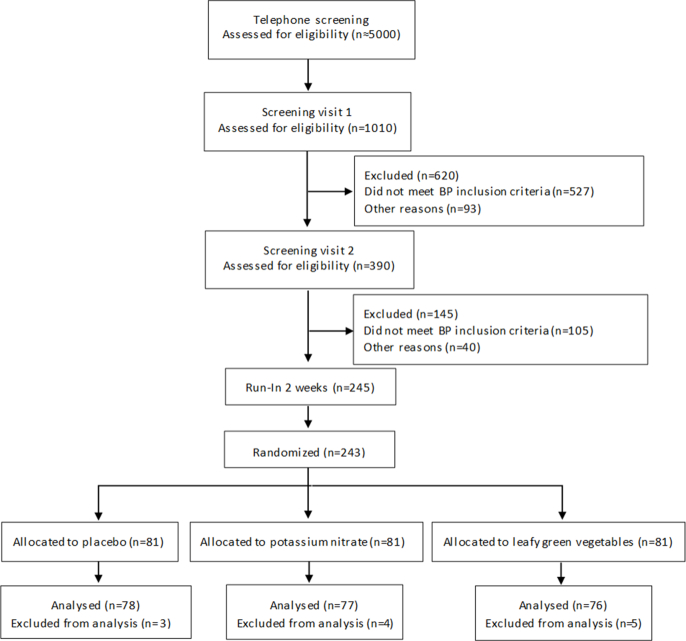
Supplemental Figure 1: Participant flow diagram: Screening, exclusions, randomization, allocation to placebo, potassium nitrate, or leafy green vegetables, and numbers analyzed.
