# Supplementary material for: A clinical study examining the effects of dietary nitrate on urinary N-nitrosamines
Source: Am J Clin Nutr. 2026 Feb 20;123(5):101239. doi: 10.1016/j.ajcnut.2026.101239 (PMC13197896; doi:10.1016/j.ajcnut.2026.101239)
Supplement: multimedia component 1 [file mmc1.docx]

**Supplemental Table 1. Baseline characteristics of the study groups**

|  | Placebo  (n=78) | Potassium nitrate  (n=77) | Leafy green vegetables  (n=76) |
| --- | --- | --- | --- |
| Age, y | 63 ± 5.6 | 62 ± 5.5 | 63 ± 5.2 |
| Female, n | 42 | 38 | 42 |
| Weight, kg | 75.8 ± 14.2 | 79.5 ± 13.9 | 76.8 ± 13.5 |
| BMI, kg/m² | 25.4 ± 3.6 | 26.6 ± 3.6 | 26.1 ± 3.1 |
| Ambulatory BP, mmHg |  |  |  |
| – SBP | 130.9 ± 9.7 | 133.2 ± 10.9 | 129.8 ± 10.3 |
| – DBP | 78.0 ± 6.4 | 79.4 ± 7.1 | 77.9 ± 7.2 |
| BP medication, n | 34 | 30 | 39 |

Notes: Values are mean ± SD. BMI-Body Mass Index, BP-Blood Pressure, SBP-Systolic Blood pressure, DBP-Diastolic Blood Pressure. Data from ref 17.
